# Rec. ST6Gal-I variants to control enzymatic activity in processes of *in vitro *glycoengineering

**DOI:** 10.1186/1753-6561-7-S6-P110

**Published:** 2013-12-04

**Authors:** Alfred M Engel, Harald Sobek, Michael Greif, Sebastian Malik, Marco Thomann, Christine Jung, Dietmar Reusch, Doris Ribitsch, Sabine Zitzenbacher, Christiane Luley, Katharina Schmoelzer, Tibor Czabany, Bernd Nidetzky, Helmut Schwab, Rainer Mueller

**Affiliations:** 1 Professional Diagnostics, Roche Diagnostics GmbH, 82372 Penzberg, Germany; 2 Pharma Biotech Development, Roche Diagnostics GmbH, 82372 Penzberg, Germany; 3 Applied Science, Roche Diagnostics GmbH, 82372 Penzberg, Germany; 4 ACIB GmbH, 8010 Graz, Austria; 5 Institute of Biotechnology and Biochemical Engineering, Graz University of Technology, 8010 Graz, Austria; 6 Institute of Molecular Biotechnology, Graz University of Technology, 8010 Graz, Austria

## Background

Glycosylation is an important posttranslational modification of proteins influencing protein folding, stability and regulation of the biological activity. The sialyl mojety (sialic acid, 5-N-acetylneuramic acid) is usually exposed at the terminal position of N-glycosylation and therefore, a major contributor to biological recognition and ligand function, *e.g*. IgG featuring terminal sialic acids were shown to induce less inflammatory response and increased serum half-life.

The biosynthesis of sialyl conjugates is controlled by a set of sugar-active enzymes including sialyltransferases which are classified as ST3, ST6 and ST8 based on the hydroxyl position of the glycosyl acceptor the Neu5Ac is transferred to [1]. The ST6 family consists of 2 subfamilies, ST6Gal and ST6GalNAc. ST6Gal catalyzes the transfer of Neu5Ac residues to the hydroxyl group in C6 of a terminal galactose residue of type 2 disaccharide (Galβ1-4GlcNAc).

To our knowledge, the access to recombinant ST6Gal-I for therapeutic applications is still limited due to low expression and/or poor activity in various hosts (*Pichia pastoris*, *Spodoptera frugiperda *and *E. coli*).

The present study describes the high-yield expression of two variants of human beta-galactoside alpha-2,6 sialyltransferase 1 (ST6Gal-I, EC 2.4.99.1; data base entry P15907) by transient gene expression in HEK293 cells with yields >100 mg/L featuring distinct mono- (G2+1SA) as well as bi- (G2+2SA) sialylation activity.

## Materials and methods

Two N-terminally truncated fragments of human ST6Gal-I (delta89, residues 89-406, and delta108, residues 109-406) were designed for transient gene expression (TGE): Instead of the natural leader sequence and N-terminal residues, both ST6Gal-I coding regions harbor the Erythropoietin (EPO) signal sequence in order to ensure correct processing of the polypeptides by the secretion machinery. Following cloning into pM1MT, expression of the ST6Gal-I coding sequences is under control of a hCMV promoter followed by an intron A.

Sialyltransferase assays: 1. Asialofetuin was used as acceptor and CMP-9F-NANA as donor substrate. Enzymatic activity was determined by measuring the transfer of 9F-NANA to asialofetuin. 2. Recombinant humanized IgG1 and IgG4 monoclonal antibodies (mabs), characterized as G2+0SA, as well as desialylated EPO were used as targets in sialylation experiments (30 μg enzyme/300 μg target protein). Both enzyme variants of ST6Gal-I (delta89 and delta108) were used under identical reaction conditions and the sialylation status was analyzed by mass spectrometry.

## Results

In using the suspension-adapted human embryonic kidney (HEK) 293-F cell line, a modified serum-free FreeStyle™ medium platform plus transfection by the 293-Free™ reagent, we were able to install a TGE shaker fermentation process with product yields of up to 200 mg/L culture supernatant. Both variants delta89 and delta108 could be isolated to >98% purity by a simple 2-step purification protocol.

**Figure 1:**
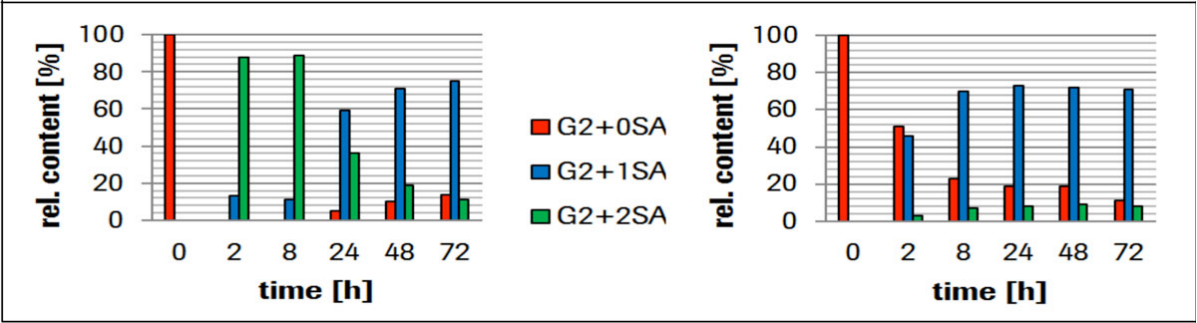
**Left panel: Variant delta89 yields 88% G2+2SA sialylation**. However, after prolonged incubation (24 hrs), the bi-sialylation is reduced to a stable mono-sialylation product, presumably by a sialydase activity. **Right panel: **Variant delta108 yields 70% G2+1SA and 7% G2+2SA sialylation.

To our surprise, both variants show a distinct and different sialylation activity as shown by sialylation kinetics of a IgG4 molecule (Figure 1).

Recently, the crystal structure of the delta89 variant could be determined as first human ST6Gal-I by SIRAS phasing using an iodide soak as derivative I [2]: An elongated glycan from a crystallographic neighbour binds to the active site, mimicking a substrate complex. An analysis of substrate interactions and comparison to other sialyltransferases allows modelling of a Michaelis complex and conclusions on the catalytic mechanism.

Due to their high expression rates and easy purification, both recombinant variants (delta89 and delta108) of human ST6Gal-I are available in large quantities and high purity. Both variants are active with high molecular weight substrates like monoclonal antibodies. To our surprise, they show different performance in sialylation experiments using with bi-antennary glycans such as mabs as well as tetra-antennary glycans (data not shown) as substrate. Under identical reaction conditions, bi-sialylated glycans are obtained in using variant delta89, whereas delta108 yields mono-sialylated glycans.

Our findings on variant delta108 are in contradiction to previous studies [3] claiming that the conserved QVWxKDS sequence, residues 94-100 of human ST6Gal-I, being essential for its catalytic activity.

To our knowledge, these human ST6Gal-I variants are the first enzymes available in large quantities and currently, recombinant alpha-2,3 sialyltransferase 1 (ST3Gal-I) and beta-1,4 galactosyltransferase 2 (B4Gal-T2) are developed in order to strengthen this enzyme portfolio. Together with the already available donor substrates (activated sugars), a complete set of reagents will be soon available for the commercial glycoengineering of proteins.

## References

[B1] Weijers CA Franssen MC Visser GM Glycosyltransferase-catalyzed synthesis of bioactive oligosaccharides Biotechnol Adv 2008 7 436 456 18565714 10.1016/j.biotechadv.2008.05.001

[B2] Kuhn B Benz J Greif M Engel AM Sobek H Rudolph MG Crystal structure of human 2,6 sialyltransferase reveals mode of binding of complex glycans Acta Crystallographica 2013 7 1826 1838 10.1107/S0907444913015412 23999306

[B3] Donadio S Dubois C Fichant G Roybon L Guillemot JC Breton C Ronin C Recognition of cell surface acceptors by two human alpha-2,6-sialyltransferases produced in CHO cells Biochimie 2003 7 311 321 12770770 10.1016/s0300-9084(03)00080-4

